# Characteristics of Interstitial Pneumonia With Autoimmune Features (IPAF): Protocol for a Multicenter Prospective Study

**DOI:** 10.2196/44802

**Published:** 2023-11-17

**Authors:** Patrycja Rzepka-Wrona, Szymon Skoczyński, Wojciech J Piotrowski, Ewa Jassem, Dariusz Ziora, Adam Barczyk

**Affiliations:** 1 Department of Pneumonology School of Medicine in Katowice Medical University of Silesia Katowice Poland; 2 Department of Lung Diseases and Tuberculosis Faculty of Medical Sciences in Zabrze Medical University of Silesia Katowice Poland; 3 Department of Pneumology Medical University of Lodz Łódź Poland; 4 Department of Pneumonology and Allergology Medical University of Gdansk Gdańsk Poland

**Keywords:** IPAF, interstitial pneumonia with autoimmune features, CTD, connective tissue disease, interstitial lung disease, idiopathic interstitial pneumonia, lung disease, pneumonia, respiratory, radiology

## Abstract

**Background:**

“Interstitial lung disease” (ILD) is a broad term encompassing diseases of different backgrounds. “Interstitial pneumonia with autoimmune features” (IPAF) is a recent term that implies the presence of autoimmunity.

**Objective:**

This study aims to determine the characteristics of Polish patients with IPAF, compare them with patients with other interstitial pneumonias, and search for the prognostic and diagnostic biomarkers of IPAF in serum and bronchoalveolar lavage fluid (BALF).

**Methods:**

This multicenter prospective study plans to recruit 240 participants divided into 1 study group and 2 control groups. Biological fluid samples will be collected according to Polish Respiratory Society management guidelines and stored at –80°C for further tests. Prospective 5-year observations of 60 newly diagnosed individuals are planned. The study will be divided into subsections. First, we plan to characterize Polish patients with IPAF (study group) against their peers with other ILDs (2 control groups). Control group 1 will comprise patients with idiopathic ILDs, including mainly idiopathic pulmonary fibrosis and nonspecific interstitial pneumonia. Control group 2 will comprise patients with connective tissue disease–associated interstitial lung diseases, such as rheumatoid arthritis, systemic sclerosis, polymyositis, dermatomyositis, Sjögren’s syndrome, mixed connective tissue disease, and systemic lupus erythematosus. Radiological and functional parameters will be analyzed. Patients will be compared in terms of high-resolution computed tomography results, the 6-minute walking test performance, and pulmonary function test parameters. The diagnosis of IPAF will be reassessed on a regular basis through multidisciplinary discussion in order to determine its clinical stability. In the laboratory arm, inflammation and fibrosis pathways will be assessed. Cytokine levels (interleukin 8, transforming growth factor beta 1, chemokine C-C motif ligand [CXCL]18, CXCL1, surfactant protein [SP]-A, SP-D, Krebs von den Lungen-6 protein, and chitinase 1) will be measured in serum and BALF. A comparative analysis of serum and BALF cytokine levels will be performed in order to establish potential differences between systemic and local inflammatory pathways. In the quality of life (QoL) arm of the study, dyspnea and cough and their impact on various aspects of the QoL will be assessed. Depression and anxiety will be measured with the Hospital Anxiety and Depression Modified Scale and the 9-item Patient Health Questionnaire, and potential correlations with symptom prevalence will be assessed.

**Results:**

This study will start recruiting patients to phase 1 in October 2023. The final results will be available in 2028. We plan to publish preliminary results after 2-3 years from the start of phase 1.

**Conclusions:**

This study will be a step toward a better understanding of IPAF etiopathogenesis and outcomes.

**International Registered Report Identifier (IRRID):**

PRR1-10.2196/44802

## Introduction

### Background

Interstitial pneumonia with autoimmune features (IPAF) was defined in 2015 by the Working Group of the European Respiratory Society (ERS) and the American Thoracic Society (ATS) as interstitial pneumonia with some clinical or serological or both types of features suggesting the presence of an underlying autoimmune disorder. However, no diagnostic criteria for any autoimmune disease are met in these patients during thorough rheumatological workup [1].

“Interstitial lung disease” (ILD) is a broad term encompassing a varied group of disorders. This includes idiopathic interstitial pneumonias (IIPs) and ILDs stemming from environmental exposure or occurring during the course of systemic diseases [2,3].

Data regarding the etiopathogenesis and natural course of IPAF are still lacking. As this is a diagnosis at the crossroads of pulmonology and rheumatology, retrospective and optimally prospective observational studies may cast more light on this subject. Retrospective studies suggest that survival in IPAF does not substantially differ from that in connective tissue disease–associated interstitial lung disease (CTD-ILD) or idiopathic pulmonary fibrosis (IPF), and prospectively, merely 10% of patients with IPAF are reclassified as having CTD-ILD, mostly on the myositis spectrum [4].

Another clinical challenge is in establishing a subset of clinical traits associated with the development of progressive interstitial fibrosis and the loss of lung function. Another retrospective study [5] on individuals with IPAF and CTD-ILD revealed that yearly decline in the forced vital capacity (FVC) is significantly more pronounced in IPAF than in CTD-ILD. Progression is influenced by the presence of anti–Sjögren’s syndrome–related antigen A (anti-SSA) antibodies and postexercise pulse increase in the 6-minute walking test (6MWT) [5].

Most available analyses of the ILD-related quality of life (QoL) and the influence of respiratory and psychological symptoms focus on the most prevalent entities (eg, IPF or sarcoidosis). Research on QoL evaluation in IPAF and its comparison with other ILDs is still scarce. Individuals with chronic disease often present with multiple symptoms, which results in decreased functioning and high medical and societal costs [6]. Depression and anxiety also contribute to the burden of chronic disease.

### Objectives

**Figure 1:**
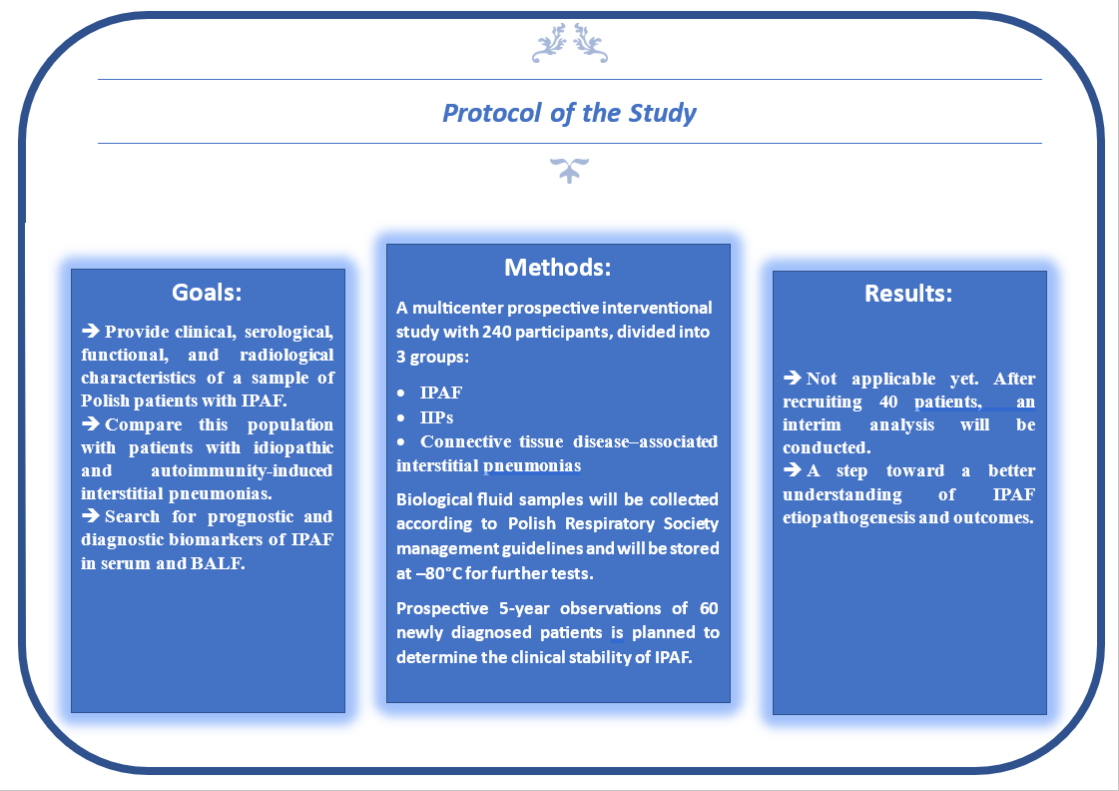
Study protocol. BALF: bronchoalveolar lavage fluid; IIP: idiopathic interstitial pneumonia; IPAF: interstitial pneumonia with autoimmune features.

The primary purpose of this study is to determine the incidence of IPAF in comparison with IIPs and ILDs occurring during the course of autoimmune diseases in 6 Polish pulmonological centers. Further objectives of this study include determination of the clinical, serological, functional, radiological, and histopathological characteristics of patients with IPAF; analysis of diagnostic strategies for specific IPAF subgroups; determination of the potential diagnostic, predictive, and prognostic features of IPAF; and prospective 5-year assessment of patients with IPAF in order to determine the stability of the diagnosis and potential progression to other diseases (eg, CTDs). Please see Figure 1 for the study protocol. To the best of our knowledge, this is the first prospective study focused on patients with IPAF, CTD-ILD, and IIP in Eastern Europe.

## Methods

### Study Population

Inclusion criteria are as follows: individuals with ILDs, aged ≥18 years, and able to provide informed consent.

The study group will comprise patients with IPAF defined according to the criteria established by the Working Group of the ERS and the ATS [1]. Control group 1 will comprise patients with the following CTD-ILDs: rheumatoid arthritis (RA), systemic sclerosis (SSc), polymyositis (PM), dermatomyositis (DM), Sjögren’s syndrome (SS), mixed connective tissue disease (MCTD), and systemic lupus erythematosus (SLE) diagnosed according to the diagnostic criteria issued by the European League Against Rheumatism (EULAR) or the American College of Rheumatology (ACR). Control group 2 will comprise patients with the following IIPs: IPF, nonspecific interstitial pneumonia (NSIP), cryptogenic organizing pneumonia (COP), acute interstitial pneumonia (AIP), respiratory bronchiolitis–associated interstitial lung disease (RB-ILD), desquamative interstitial pneumonia (DIP), and lymphocytic interstitial pneumonia (LIP). Previously untreated patients with a new diagnosis of ILD were recruited for a pilot study (a separate population of patients) [7].

Exclusion criteria include age<18 years; respiratory tract infection, including minor upper respiratory tract infections (eg, the common cold) 4 weeks prior to the diagnostic process; asthma; tuberculosis; asbestosis; chronic hypersensitivity pneumonia; Hermansky-Pudlak syndrome; and other causes of genetic ILDs (eg, lymphangioleiomyomatosis, tuberous sclerosis complex [TSC], Birt-Hogg-Dubé syndrome, dyskeratosis congenita, familial forms of ILD, immunodeficiency syndromes); a history of therapy for ILD, including systemic glucocorticosteroids (GCs; both pulsed and long-term low-dose therapy); pregnancy and nursing; malignancy; HIV seropositivity; and a lack of ability to provide informed consent. For the QoL arm of the study, we will also exclude participants unable to communicate verbally.

### Ethical Considerations

The study will be carried out according to the Code of Ethics of the World Medical Association (Declaration of Helsinki). This protocol was reviewed and approved by the Ethical Board Committee of the Medical University of Silesia (decision no. KNW/0022/KB1/130/18/19). The trial is registered with reference number NCT03870828. All participants will sign the informed consent form prior to recruitment and will be informed of their right to waive consent at any time without a negative effect on their care. The informed consent form was reviewed and accepted by the Ethical Board Committee in terms of informativeness and understandability. The form allows for clinical data collection and secondary analysis. A copy of the form will be provided to each participant.

The study team will explain data analysis, the study duration, and objectives to the participants. We find it worth mentioning that in addition to drawing larger volumes of blood, storing biological fluids, and completing the questionnaires, no procedures beyond standard care will be performed. Therefore, participants will not be burdened with additional risks or prolongation of hospitalization/outpatient clinic visits. Study data will be deidentified to protect the privacy of participants. Data confidentiality will be maintained. No monetary compensation will be given to the participants. Voluntary participation and the time of the participants will be respected.

### Study Design

#### Phase 1

All patients meeting the inclusion criteria and not meeting the exclusion criteria regardless of prior treatment history will be recruited to phase 1 of the study. The recruitment process duration is estimated at 12 months. The previous recruitment of 40 patients led to a pilot study [7], which impacted further study design. The maximum number of patients included in this phase will be 80 (33.3%) in the IPAF group, 80 (33.3%) in the CTD-ILD group, and 80 (33.3%) in the IIP group.

#### Phase 2

Only patients with newly diagnosed disease (eg, no prior diagnosis of ILD and, hence, no prior treatment) will be recruited. The estimated recruitment duration is 36 months. The number of patients to be included in this phase will be 20 (33.3%) in the IPAF group, 20 (33.3%) in the CTD-ILD group (only those with RA, SSc, PM/DM), and 20 (33.3%) in the IIP group (only those with IPF, NSIP, COP, LIP; optimally 10, 50%, patients in the IPF subgroup and 10, 50%, in the non-IPF subgroup). Patients who were previously recruited to phase 1 of the study and meet the inclusion criteria of phase 2 may also be included.

**Figure 2:**
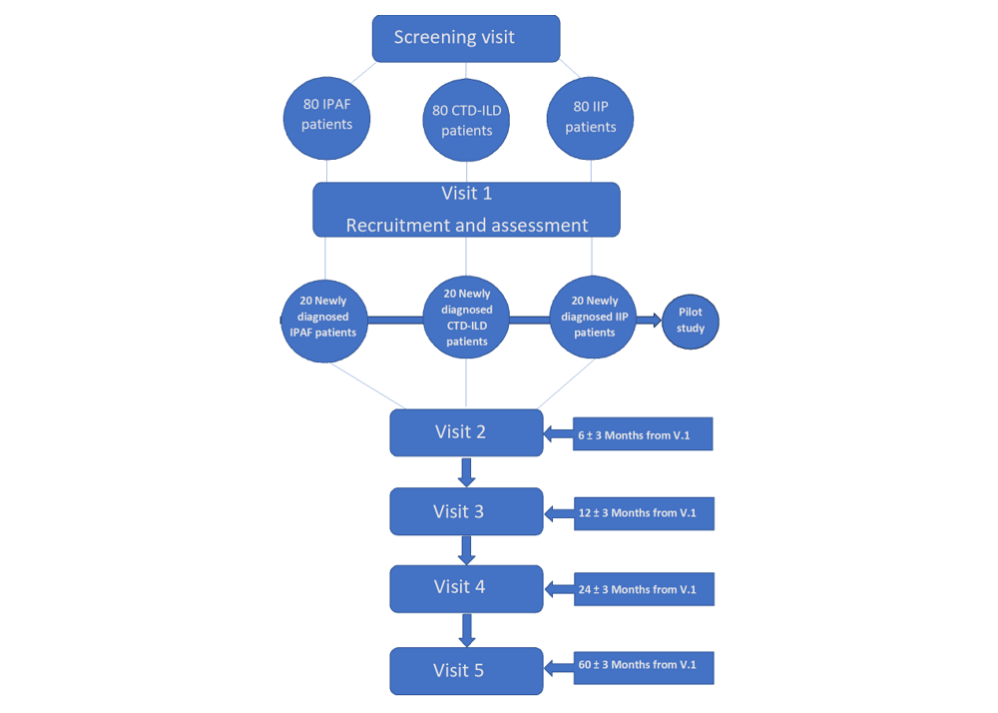
Flowchart of the study design. CTD-ILD: connective tissue disease–associated interstitial lung disease; IIP: idiopathic interstitial pneumonia; IPAF: interstitial pneumonia with autoimmune features; V: visit.

Please see Figure 2 for a flowchart of the study design.

#### Visit 1

A thorough medical history will be obtained using the Visit 1 questionnaire. During the first hospitalization, the following procedures will be performed:

- Pulmonary function tests (PFTs)
- Depression and anxiety assessment
- Blood analysis
- Fiberoptic bronchoscopy (FOB) with bronchoalveolar lavage (BAL) and bronchial mucosa sampling
- The 6MWT
- Computed tomography (CT)
- Transthoracic echocardiography (TTE)

#### Visit 2

Only patients who qualify for phase 2 of the study will have further visits (60 newly diagnosed patients). The following procedures will be performed after an average of 6 (SD 3) months from visit 1:

- The Visit 2 questionnaire
- Cough assessment
- Dyspnea assessment
- Depression and anxiety assessment
- PFTs, the 6MWT, and oxygen saturation (SpO2) measurement
- Arterial blood gas (ABG) analysis (if indicated)

#### Visit 3

The following procedures will be performed after an average of 12 (SD 3) months from visit 1:

- The Visit 2 questionnaire
- Cough assessment
- Dyspnea assessment
- Depression and anxiety assessment
- PFTs and the 6MWT
- High-resolution computed tomography (HRCT)
- Blood sampling (including testing for autoantibodies and potential biomarkers)
- FOB with BAL (optionally, if clinically indicated)
- Capillary blood gas (CBG) analysis
- TTE
- Rheumatological consultation

#### Visit 4

The following procedures will be performed after an average of 24 (SD 3) months from visit 1:

- The Visit 2 questionnaire
- Cough assessment
- Dyspnea assessment
- Depression and anxiety assessment
- PFTs and the 6MWT
- HRCT (if clinically indicated)
- Blood sampling (including testing for autoantibodies and potential biomarkers)
- FOB with BAL (if clinically indicated)
- CBG analysis

#### Visit 5

The following procedures will be performed after an average of 60 (SD 3) months from visit 1:

- The Visit 2 questionnaire
- Cough assessment
- Dyspnea assessment
- Depression and anxiety assessment
- PFTs and the 6MWT
- HRCT (if clinically indicated)
- Blood sampling (including testing for autoantibodies and potential biomarkers)
- FOB with BAL (if clinically indicated)
- CBG analysis
- Rheumatological consultation

### Diagnostic Procedures

#### Pulmonary Function Tests

PFTs will include spirometry with a reversibility test (in the case of obstruction), body plethysmography, and diffusion capacity for carbon monoxide (DLCO). Both absolute and predicted values will be recorded: forced expiratory volume in 1 second (FEV_1_), FVC, vital capacity (VC), FEV_1_/VC ratio, total lung capacity (TLC), diffusing capacity for carbon monoxide measured by the single-breath method (DLCO SB), inspiratory thoracic gas volume (ITGV), and residual volume/total lung capacity (RV/TLC) ratio. Cough assessment will be conducted using the Leicester Cough Questionnaire and numeric scale. Dyspnea assessment will be performed using the Likert scale and the Modified Borg Scale.

#### Depression and Anxiety

Depression and anxiety assessment will be conducted using the Hospital Anxiety and Depression Modified Scale (HADS-M) and the 9-item Patient Health Questionnaire (PHQ-9).

#### Blood Analysis

Briefly, a vein will be punctured in order to take a blood sample, and 30 mL of blood will be drawn. Next, 15 mL of plasma and 15 mL of full blood will be stored separately at –80°C. The following will be analyzed: N-terminal prohormone of brain natriuretic peptide (NT-proBNP) biomarkers routinely used during rheumatological workup and potential IPAF biomarkers. For this, 15 mL of venous blood will be drawn and divided into 2 parts (10 mL and 5 mL), and no anticoagulant will be added to the samples.

The first sample (10 mL) will be sent to the local laboratory for routine analysis of NT-proBNP and rheumatological factor (RF) and for anti–cyclic citrullinated peptide (anti-CCP) and antinuclear antibody (ANA) screening tests. In the case of the confirmed presence of ANAs and the determination of a fluorescence pattern (eg, homogenous, speckled, peripheral, nucleolar), further tests will be ordered: anti–double-stranded DNA (anti-dsDNA), anti-Smith (anti-SM), anti-SSA/Ro, anti-SSB/La, anti–topoisomerase I (anti-Scl70), and antiribonucleoprotein (anti-RNP) antibodies and myositis-specific autoantibodies, such as anti–histidyl-tRNA synthetase (anti-Jo1), anti–threonyl-tRNA synthetase (anti-PL7), anti–alanyl-tRNA synthetase (anti-PL12), anti–Mi2 antigen (anti-Mi2), anti–human exosome complex (anti-PmScl), and anti–melanoma differentiation–associated 5 (anti-MDA5) antibodies.

From the second sample of 5 mL, after clot formation, approximately 2.5 mL of serum will be separated and then divided into 5 portions of 0.5 mL each and placed in Eppendorf tubes. The samples will be marked according to a system allowing for patient anonymization: medical center symbol (sample number - type of biological material - date of sample collection, eg, A12-S_10/04/18). The samples will be stored at –70°C. They will be then used for IPAF biomarkers’ identification. This includes testing for chemokine C-C motif ligand 18 (CXCL18), surfactant protein (SP)-A, SP-D, Krebs von den Lungen-6 protein (KL-6), and chitinase 1 (CHIT1).

For genetic testing, 15 mL of full blood will be collected and divided into 10 Eppendorf tubes containing 1.5 mL of blood each. Samples will be marked according to the aforementioned organizational pattern and stored at –80°C.

#### Fiberoptic Bronchoscopy With Bronchoalveolar Lavage and Bronchial Mucosa Sampling

Before FOB, local anesthesia with lidocaine and conscious sedation with midazolam and optionally fentanyl will be performed according to detailed anesthesia protocols used in the endoscopy units of the respective medical centers taking part in the study. An intravenous cannula will be inserted prior to the procedure. During the endoscopy, the patient will be monitored according to safety protocols effective in the respective endoscopy units.

Bronchoalveolar lavage fluid (BALF) will be obtained from areas characterized by the greatest interstitial fibrosis involvement on HRCT. If such an area cannot be distinguished, BALF will be obtained from the middle lobe bronchus or the lingula on the side (B4, B5). Localization will be chosen based on HRCT results and will be recorded in the patients’ medical files. Next, 200 mL of sterile 0.9% saline solution, previously warmed to body temperature, will be inserted in parts through a 25 mL syringe to perform BAL. For the test to be deemed diagnostic, the protocol will target BALF salvage of at least 60%.

BAL will be performed according to the guidelines of the ERS [8] and the practical guidelines of the Polish Respiratory Society [9]. A differential cell count will be performed in every sample. BALF samples will be processed by an experienced laboratory technician based on Polish Respiratory Society guidelines [9].

Microscopic slide preparation, determination of cell viability, and determination of the dominant population will be performed according to standard laboratory procedures in the Polish Respiratory Society guidelines. The whole-cell count and cell viability will be measured in a sample derived from fluid that has undergone filtration or the first centrifugation. Samples will be prepared with the cytospin method (routinely 1200-2000 rpm for 10 minutes), and cellular smears will be assessed in cytospin samples stained according to the May-Grünwald-Giemsa method.

BALF samples will be secured for further tests, including testing for potential IPAF biomarkers. Briefly, 15 mL of BALF will be divided into 10 Eppendorf tubes containing 1.5 mL of BALF each and stored at –70°C. Low-speed centrifugal supernatants of BALF will be obtained from each sample. We will measure the concentration of potential biomarkers in each sample using the sandwich enzyme-linked immunosorbent assay (ELISA) technique.

Next, 5 mucosa samples will be obtained from the middle lobe bronchus for histopathological assessment later. Directly after sample collection, they will be put into dry Eppendorf tubes and frozen at –80°C. In addition, transbronchial lung cryobiopsy may be used to obtain material for histopathological assessment.

#### The 6-Minute Walking Test

The submaximal exercise 6MWT, which entails the measurement of the distance walked over a span of 6 minutes, will be performed according to the detailed protocol used in the respective medical centers. The blood pressure (BP), heart rate (HR), and SpO_2_ will be measured directly before and after the test. The following will be recorded: whether the patient completed the test, the distance walked (m), SpO_2_, the SpO_2_ nadir, and dyspnea sensation measured with the Modified Borg Scale and the Visual Analogue Scale (VAS). If the test is stopped or paused before 6 minutes, the reason (eg, dyspnea, high BP, cardiac arrythmia, limb pain, angina, or other) will be recorded.

The 6MWT will be performed according to Polish Respiratory Society guidelines [10]. Participants will be informed at 30-second intervals about the amount of time remaining. The predicted distance will be calculated based on the equation published in Polish Respiratory Society guidelines for the interpretation of the 6MWT as the most accurate for participants’ characteristics [10].

Pulse oximetry will be performed for all patients. If SpO_2_<92% or there are clinical indications for oxygen therapy, ABG analysis will be performed. For ABG analysis, an artery (radial or femoral) is punctured in order to take a sample of arterial blood. The artery is then compressed in order to prevent bleeding/hematoma. The procedure is performed according to ERS guidelines [11].

#### Computed Tomography

HRCT of the lung will be performed during hospitalization. If the patient provides a CT scan from up to 6 months prior to recruitment (eg, from the outpatient clinic), we will analyze both scans and the radiological descriptions. To ensure the patient’s anonymity, a CD containing the patient’s scans will be marked by the technician according to the aforementioned pattern. Radiological analysis will be performed using the OsiriXLite DICOM Viewer. The following densitometric values will be measured: mean lung attenuation (MLA), kurtosis, skewness, and deviation of lung radiodensity (SD_IR_). Statistical analysis of the data will be performed using Statistica software. Data will be presented as the median value and IQRs. Differences between quantitative data will be analyzed using the Kruskal-Wallis test and the post hoc Dunn test.

#### Transthoracic Echocardiography

TTE with a thorough right heart description will be performed. The following parameters will be noted in the patient’s medical file: main pulmonary artery diameter; acceleration time; pulmonary valve peak velocity, tricuspid annular peak systolic velocity (TAPSV), tricuspid annular plate systolic excursion (TAPSE), and tricuspid valve peak regurgitation velocity; right ventricular basal and right ventricular midcavity diameters; right ventricular outflow tract and proximal and distal right ventricular outflow; right atrial short- and long-axis, inferior vena cava, and superior vena cava diameters; and right ventricular wall thickness.

Rheumatological consultation will be provided to patients with IPAF and CTD-ILD to verify the diagnosis.

#### Choice of Candidate IPAF Biomarkers

KL-6 (mucin 1) is a glycoprotein encoded by the *MUC1* gene and is expressed on the outer surface of type II alveolar epithelial cells and the airway epithelium. It has been proposed as a biomarker of IPF [12]. KL-6 probably promotes fibroblasts’ migration, proliferation, and survival in the respiratory system [12]. High serum KL-6 levels have been found in patients with IPF, NSIP, and other ILDs [13-15], which is associated with an aggressive clinical course and could also serve as a predictor of acute exacerbation [16]. High KL-6 levels have also been found in the BALF of individuals with various ILDs, including IPF [17].

Another proposed ILD activity biomarker is CC-chemokine ligand (CCL)18. In a population of patients with idiopathic ILD and SSc, it was revealed that the BALF CCL18 level was negatively correlated with the TLC and the DLCO and positively correlated with BALF neutrophil and eosinophil cell counts. During the follow-up period of ≥6 months, a negative correlation was observed between the decrease in the predicted TLC and the increase in serum CCL18 levels [18]. We hypothesize that such correlation also exists in IPAF, and we will test the theory that patients with IPAF with autoantibodies associated with SSc (anti-PmScl) have higher serum or BALF CCL18 levels than those who test negative for these autoantibodies.

Surfactant proteins SP-A and SP-D are large hydrophilic molecules produced by Clara cells and type II alveolar epithelial cells. SP-A and SP-D play an important role in innate immunity and modulate the excessive inflammatory response on the alveolar surface [19-21]. SP-A and SP-D levels are higher in patients with IPAF than in healthy individuals, patients with infectious pneumonia, or patients with nonfibrotic lung diseases [22-24]. Moreover, serum SP-D levels are lower in the IIP non-IPF group than in the IPAF group [22]. SP-A levels cannot be used to differentiate between patients with IPAF and CTD-ILD [24].

According to Xue et al [22], a negative correlation of serum SP-A levels and the DLCO was revealed in the IIP group (which included 27/69 patients with IPAF, although this subgroup was not separately analyzed). A negative correlation was also revealed with FEV_1_ and the FVC [22]. This conclusion was partially confirmed by Wang et al [23], who revealed a negative correlation between serum SP-A levels and changes in the DLCO, FEV_1_, and FVC values after treatment. Additionally, Wang et al [23] suggested a prognostic role of SP-A: the pretreatment levels were found to be significantly lower than the posttreatment ones in individuals with progressive fibrosis during the course of IPAF. Moreover, a positive correlation was found between shifts in KL-6 and SP-A levels [23]. In a prospective study, Xue et al [24] observed a significant difference in serum SP-A levels measured at baseline and after 1-year follow-up. In individuals whose condition had exacerbated, SP-A levels were correlated with lung involvement scores on HRCT [24].

These findings need to be validated in European populations. There is a need to determine whether there is a correlation between lung involvement and a decrease in pulmonary function and SP-A/SP-D levels in the bodily fluids of patients with IPAF.

CHIT1 is a member of the glycosyl hydrolase family 18. It is expressed predominantly in polarized and differentiated macrophages and acts as an innate immune mediator digesting cell walls of pathogens that contain chitin (eg, fungi). CHIT1, due to its dysregulated activity, is a proposed marker in granulomatous and fibrotic ILDs associated with interstitial inflammation and lung tissue remodeling.

A study by Bargagli et al [25] revealed elevated CHIT1 activity in the BALF of individuals with IPF in comparison with the healthy control group; however, no significant differences in the serum levels were noted. This suggests the role of CHIT1 expression in interstitial macrophages in tissue injury and remodeling. We hypothesize that CHIT1 may play an important role in the pathogenesis of other ILDs, including IPAF, especially with a progressive fibrosing phenotype.

Lee et al [26] revealed that in 2 different cohorts of individuals with SSc, CHIT1 activity and concentrations increased in the serum and BALF compared to demographically matched healthy controls. Individuals with SSc-ILD, in comparison with patients without lung involvement, were characterized by higher CHIT1 serum activity, which was also correlated with disease severity. The authors revealed that CHIT1 upregulates transforming growth factor beta 1 (TGF-β1) receptor expression and its signaling in fibroblasts, which makes CHIT1 a modulator of progressive interstitial lung fibrosis [27].

These findings raise the question whether CHIT1 or TGF-β1 and their interaction could be a potential target for SSc pharmacological therapy. As the etiopathogenesis of IPAF is still obscure, we plan to analyze both circulatory and lung activities of the enzyme to establish its potential role in the disease pathogenesis.

S100A9, also referred to as myeloid-related protein 14 or calgranulin B, is a member of the calcium-binding protein S100 family and is mostly expressed in neutrophils but also on vascular endothelial cells and mature macrophages. S1009A expression is enhanced by some chronic inflammatory factors [28]. It acts as an immunomodulatory, proinflammatory, and profibrotic agent [29]. It is mostly secreted in inflammatory lesions [30], and it has been suggested as a potential target for IPF treatment [31] and as a candidate biomarker to differentiate between IPF and other fibrotic ILDs [32]. However, convincing evidence from contemporary trials is yet scarce.

Elevated serum S100A9 levels have been reported in samples obtained from patients with RA, SLE, SSc, vasculitis, lung cancer, and various other diseases associated with chronic inflammation [33-35]. It has also been demonstrated that S1009A may promote atherosclerosis [36]. In an observational study by Lin et al [37], it was revealed that S100A9 levels were significantly elevated in BALF samples obtained from patients with severe IPF. It has also been demonstrated that S100A9 levels in the BALF of patients with IPF are positively correlated with the percentage of BALF neutrophils. This suggests a relationship between S1009A levels and disease activity. Some studies have reported that BALF S100A9 levels are significantly elevated in individuals with pulmonary involvement during the course of SSc [38,39].

CCL2 is a protein that attracts T lymphocytes, natural killer lymphocytes, and fibrocytes [40]. It is associated with inflammatory processes, cell migration and activation, fibrosis, and angiogenesis [41] through its 7-transmembrane-spanning G-protein-coupled CC-chemokine receptor 2 [42]. CCL2 plays a significant role in the pathogenesis of various diseases, such as tuberculosis [43], asthma [44], and RA [45].

A connection was established between CCL2 polymorphism and the occurrence of autoimmune disorders. The *CCL2* gene (–2518G/A) single-nucleotide polymorphism has been shown to be associated with sarcoidosis [46]. Increased CCL2 levels have been found in the serum and BALF of patients with IPF [47]. In vitro and in vivo studies have revealed that CCL2 is associated with fibrosis by supporting monocyte-/macrophage-mediated inflammation, angiogenesis, collagen synthesis, myofibroblast cell differentiation, and fibroblast activation [48].

It remains unclear whether the chemokine profile of serum and BALF of individuals with IPAF, especially patients with progressive fibrosis, resembles that of patients with IPF.

### Data Collection

Data will be collected via questionnaires: the Leicester Cough Questionnaire, St. George’s Respiratory Questionnaire, the Fatigue Assessment Score and 36-item Short Form (SF-36) v.2 Quality of Life Questionnaire, the HADS-M, and the PHQ-9. To help unify data and due to copyright reasons, surveys for physicians will be set up on servers of the Medical University of Silesia. All the aforementioned documents will be provided to the patients in their native tongue and have been validated in the Polish population.

### Statistical Analysis

Statistical tests will be chosen due to variable distribution. To verify the normality of variable distribution, the Shapiro-Wilk test will be performed. To compare parameters between the 1 study and 2 control groups, ANOVA or the Kruskal-Wallis test will be performed (for normal or nonnormal variable distribution, respectively). To correlate the variables, Pearson or Spearman correlation tests will be performed (for normal or nonnormal variable distribution, respectively). To determine the relationship between variables, the linear regression test will be performed.

## Results

Although the study was designed and Ethical Board Committee approval granted in the past, we have experienced difficulties in the phase of patient recruitment due to the outbreak of the novel coronavirus.

It is worth noting that the majority of tests are routinely performed in the diagnostic workup of patients with ILDs; therefore, patients will not be subjected to additional risks. Discomfort may be caused by the drawing of more blood samples than usual; however, this will be reduced by the use of vacuum blood collection systems.

This study will start recruiting patients to phase 1 in October 2023. The final results will be available in 2028. We plan to publish preliminary results after 2-3 years from the start of phase 1.

## Discussion

### Summary

As IPAF is an exclusion diagnosis, we believe that in the era of genetic testing advancements, genetic disorders manifesting as ILDs should be considered as alternative etiologies. In our study, the local bioethics committee granted permission to perform genetic testing, and we plan to bank full blood, with the intention to perform such procedures.

There have been clinical reports of individuals with signal transducer and activator of transcription 3 (STAT3) gain-of-function syndrome [49] or cytotoxic T cell antigen 4 (CTL-A4) checkpoint haploinsufficiency [50], which could fulfill the 2015 criteria. Moreover, a newly discovered coatomer protein complex subunit alpha (COPA) syndrome causes progressive ILD [51]. The impaired COPA protein may contribute to endoplasmic reticulum stress and therefore trigger interferon signaling pathways and T cell–mediated inflammation [51].

Newton et al [52] revealed that the leukocyte telomere length is longer in individuals with IPAF and CTD-ILD than in those with IPF. The telomere length correlates with lung function and lung transplant outcomes in IPAF. Moreover, mucin 5B (MUC5B) and Toll-interacting protein (TOLLIP) polymorphisms are associated with both IPAF and IPF, which suggests that these 2 clinical entities have similar genetic backgrounds. Various polymorphisms are found in CTDs, such as RA, SS, and SSc, but to date, there are no data on whether the presence of these polymorphisms is associated with the development of IPAF. Hopefully, an in-depth understanding of molecular pathways could lead to the development of targeted therapies.

A practical question we are going to face stems from the fact that instructions for most ELISA kits that are available for research use in our area only contain suggestions/guidelines for serum dilution. Therefore, we will need to either predict the levels of the aforementioned proteins in BALF or experiment with dilution. As we are unsure how much time it will take to recruit the adequate number of individuals, we plan to perform a miniexperiment beforehand: First, we will test the serum according to the manufacturer’s guidelines. If the standard 1:40 dilution does not lead to the detection of predicted concentrations, we will check the 1:20 dilution, 1:5 dilution, and undiluted samples. The next step will be testing BAL samples. We will test 1:20 dilution samples, and again, if this does not result in the detection of protein concentrations within the range provided in the kit instruction, we will test the 1:5 dilution and undiluted specimens.

The clinical stability of IPAF is an important subject, as the development of mild extrapulmonary symptoms may be overlooked. In our opinion, this specific population requires above-average clinical vigilance due to the fact that reclassification as definite CTD-ILD prompts a change in the therapeutic approach based on ACR/EULAR guidelines. This is why we plan to cooperate closely with rheumatologists regarding assessment of the extrapulmonary manifestations of autoimmunity. However, there is also a possibility that an entity that had been initially classified as idiopathic ILD may later be reclassified as IPAF. To test this hypothesis, we plan periodic rheumatological revaluations of this subgroup, together with retesting for ANAs (serological domain of IPAF).

Data on the progression of IPAF to CTDs are scarce and conflicting. Scirè et al [53] reported that 42% of patients with IPAF with antisynthetase antibody positivity were ultimately reclassified as RA-ILD, PM/DM-ILD, or myositis-RA overlap syndrome. Ito et al [54] also reported that 12.2% of individuals meeting IPAF serological and morphological criteria were diagnosed with CTD during a median follow-up period of 54 months. However, Chartrand et al [55] found that none of the 56 individuals with IPAF was rediagnosed over a follow-up period of 48 months.

Another question is when to initiate treatment during the course of IPAF. Before IPAF was defined, ILD with symptoms of underlying autoimmune disorder but without extrapulmonary manifestation could have been classified as IIP and therefore have delayed treatment initiation. In our prospective observational study, we will surely face a challenge in identifying patients with IPAF who require early clinical intervention. We hope that during the 60-month observation period, we will be able to identify molecular, radiological, and functional prognostic factors, which, in the future, will help lay the foundation for IPAF therapy guidelines.

In a study on 412 patients with CTD-ILD or IPAF, Li et al [56] analyzed the relationship between these 2 entities in order to identify which individuals with IPAF should commence treatment early. They concluded that based on similar yet milder symptoms and ANA positivity, IPAF is in fact an early stage of CTD-ILD and therefore treatment should commence early. After immunosuppressive treatment, they reported an increase in the FVC and DLCO in this population. The choice of adequate pharmacotherapy is another clinical question that we will face during prospective observation.

D’Silva et al [57] presented a case series of 50 patients from 2 centers who were treated with rituximab (RTX). The majority experienced either clinical improvement or stability understood as global assessment of performance in terms of oxygen requirement, unplanned hospitalization due to respiratory reasons, and 1-year survival, despite baseline differences regarding age, comorbidities, and prior treatment history.

Although we do not plan a randomized controlled trial, in prospective observation, we will face clinical decisions regarding treatment implementation. We hypothesize that some patients will have a history of treatment with systemic GCs or cyclophosphamide (CYC), and we wonder whether the results of studies on CTD-ILDs can be extrapolated to the IPAF population. The use of CYC is associated with an increased risk of malignancy, infections, and bone marrow suppression [58].

Some patients in this study had previously used mycophenolate mofetil (MMF) or tacrolimus, which may have impacted their performance on RTX. The implementation of RTX therapy, especially in patients with refractory ILD, should be considered due to its favorable safety profile [59]. We also hypothesize that a usual interstitial pneumonia (UIP) radiological pattern, which is associated with higher mortality in patients with IPAF [60], may impact treatment efficacy.

We wonder whether studies on patients with RA-ILD who generally have a UIP pattern may be extrapolated to this specific IPAF population, and in prospective observation, we plan to exteriorize 2 subpopulations (UIP-RA and UIP-IPAF) and compare patients’ outcomes in terms of oxygen requirement, PFT results, 6MWT performance, and 1-, 3-, and 5-year survival.

We are looking forward to cooperating with rheumatologists for multidisciplinary discussion.

### Impact of Pilot Study Results on the Protocol

In our pilot study [7], we revealed a greater prevalence of gastrointestinal reflux disease (GERD) in the CTD-ILD group than in the IPAF and IIP groups. Data on the impact of GERD on fibrosis pathogenesis are conflicting. There are data suggesting that exposure of the interstitial tissue to a low pH results in repeated lesions and, consequently, progressive fibrosis. However, there are also data supporting a hypothesis that GERD actually results from progressive fibrosis and a shift in intrathoracic pressure due to altered anatomy. Therefore, we decided to launch a substudy on patients with progressive fibrosing ILD (PF-ILD), focusing on the impact of GERD on the disease course. We will monitor 24-hour impedance-pH to objectify GERD episodes and correlate them with symptoms.

In the pilot study [7], it was also revealed that BALF levels of interleukin 8 (IL-8), CXCL1, and TGF-β1 were significantly higher in the CTD-ILD group than in the IPAF and IIP groups. We hope that the analysis of 240 subjects in this full-size study will reveal more defined differences between study groups. We plan to analyze subgroups (eg, RA-ILD or SSc-ILD) and compare them with their IPAF counterparts (based on the presence of clinical symptoms and specific autoantibodies). We also plan to distinguish an IPAF subgroup associated with the presence of antisynthetase autoantibodies and compare it with the PM/DM-ILD subgroup in terms of clinical stability and outcomes.

Our pilot study [7] also revealed that the BALF IL-8 concentration is associated with the presence of skin teleangiectasias. IL-8 is a known proangiogenic factor. In this full-size study, we plan to perform videocapillaroscopy to stratify participants according to the Cutolo nailfold capillaroscopy classification and measure the concentration of proangiogenic cytokines in both serum and BALF. We will be able to compare corresponding Cutolo subgroups of SSc-ILD and IPAF and determine the impact of proangiogenic factors on pathological angiogenesis.

We also hypothesize that there will be significant intergroup differences between the reported severity of cough and dyspnea as well as psychological symptoms and their impact on various aspects of the QoL.

### Conclusion

This study will be a step toward a better understanding of IPAF etiopathogenesis and outcomes.

## Abbreviations

6MWT: 6-minute walking test
ABG: arterial blood gas
ACR: American College of Rheumatology
ANA: antinuclear antibody
anti-SSA: anti–Sjögren’s syndrome–related antigen A
ATS: American Thoracic Society
BAL: bronchoalveolar lavage
BALF: bronchoalveolar lavage fluid
CBG: capillary blood gas
CCL: CC-chemokine ligand
COP: cryptogenic organizing pneumonia
COPA: coatomer protein complex subunit alpha
CT: computed tomography
CTD-ILD: connective tissue disease–associated interstitial lung disease
DLCO: diffusion capacity for carbon monoxide
DM: dermatomyositis
ERS: European Respiratory Society
ELISA: enzyme-linked immunosorbent assay
EULAR: European League Against Rheumatism
FEV1: forced expiratory volume in 1 second
FOB: fiberoptic bronchoscopy
FVC: forced vital capacity
GERD: gastrointestinal reflux disease
HADS-M: Hospital Anxiety and Depression Modified Scale
HRCT: high-resolution computed tomography
IIP: idiopathic interstitial pneumonia
ILD: interstitial lung disease
IPAF: interstitial pneumonia with autoimmune features
IPF: idiopathic pulmonary fibrosis
LIP: lymphocytic interstitial pneumonia
MCTD: mixed connective tissue disease
NSIP: nonspecific interstitial pneumonia
PFT: pulmonary function test
PHQ-9: 9-item Patient Health Questionnaire
PM: polymyositis
QoL: quality of life
RA: rheumatoid arthritis
RB-ILD: respiratory bronchiolitis–associated interstitial lung disease
RTX: rituximab
SpO_2_: oxygen saturation
SS: Sjögren’s syndrome
SLE: systemic lupus erythematosus
SSc: systemic sclerosis
TGF-β1: transforming growth factor beta 1
TLC: total lung capacity
TTE: transthoracic echocardiography
VC: vital capacity

## Data Availability

Due to privacy reasons, anonymized data will be available upon request from the corresponding author.
